# Comparison of Diff-Quick and Spermac Staining Methods for Sperm Morphology Evaluation

**DOI:** 10.18502/jri.v24i3.13272

**Published:** 2023

**Authors:** Lincoln Bastos Farias, André Rodrigues da Cunha Barreto-Vianna, Mariana Duque de Mello, Alexandre Leseur dos Santos, Cristiane da Fonte Ramos, Paula Fontoura

**Affiliations:** 1- Rio de Janeiro Sperm Bank (Banco de Sêmen do Rio de Janeiro), Rio de Janeiro, Brazil; 2- Federal University of Rio de Janeiro, Rio de Janeiro, Brazil; 3- Federal University of Paraná-Palotina, Paraná, Brazil; 4- State University of Rio de Janeiro, Rio de Janeiro, Brazil

**Keywords:** Human, Morphology, Spermatozoa, Staining

## Abstract

**Background:**

The objective of the current study was comparing the impact of two staining techniques on semen morphological parameters and their influence on patient diagnosis. The ideal staining method should preserve cell integrity while providing detailed information.

**Methods:**

Semen samples from fifty men were stained using Diff-Quick or Spermac methods. Morphological parameters were classified based on the Tygerberg criteria, and final diagnosis was according to WHO manual guidelines. Statistical analysis was performed through conducting paired t-tests or Wilcoxon rank-sum tests, with GLIMMIX and Fisher’s exact test for determining the significance (p≤0.05).

**Results:**

Both staining methods highlighted head and tail regions, with Spermac offering better visualization of the midpiece. Spermac demonstrated fewer normal spermatozoa (2.8±0.3%) compared to Diff-Quick (3.98±0.4%; p=0.0385). Midpiece abnormalities were more evident with Spermac (55.7±2.1%) than Diff-Quick (24.8±2.0%; p<0.0001). No significant difference was found in head and tail abnormalities (p>0.05).

**Conclusion:**

Diff-Quick staining resulted in a higher proportion of normal spermatozoa, primarily due to its midpiece evaluation. The choice of staining method significantly impacts the diagnosis of infertile males. These findings have important implications for clinical practice and future research, suggesting the need for further investigations to assess different staining methods and determine optimal diagnostic thresholds.

## Introduction

Throughout the years, the minimum threshold for normal human sperm morphology has significantly declined. In the span of three decades between the publication of the first and latest manual of the World Health Organization (WHO) for the examination and processing of human semen, the evaluation of morphology has been increasingly emphasized (1). The standards have become more stringent, and the quality of the materials used for the examination has been improved (2).

The optimal staining method for evaluating sperm morphology is one that induces fewer changes and provides more details about all parts of the cell (3). The current WHO manual recommends three staining methods for morphology analysis, including Papanicolaou, Shorr, and Diff-Quick (1). Although Papanicolaou stain is a lengthy and complex procedure, it has been established as the standard method for this evaluation. However, there are several alternative methods that are simpler and faster, such as Giemsa, Testsimplets, SpermBlue, and Spermac (4). Spermac is one of the staining methods recommended in the handbook of “A Practical Guide to Basic Laboratory Andrology” (5).

Although in Brazil, the most widely used staining techniques for evaluating sperm morphology are Diff-Quick and Spermac, there is limited literature on the comparison of the accuracy of these two techniques using human semen. There are conflicting data regarding the advantage of Spermac over Diff-Quick stain in rainbow trout (6) and stallion (7) spermatozoa. This shortage of information suggests that additional studies using human semen should be conducted, which could significantly contribute to a better standardization of sperm morphology evaluation and a more accurate diagnosis.

The objective of the current study was to evaluate the semen morphological parameters after the application of the Diff-Quick and Spermac staining techniques and to assess whether the staining technique has an impact on the patient’s diagnosis or not.

## Methods

### Sperm collection:

Semen samples were collected from fifty men visiting the sperm bank in Rio de Janeiro, Brazil, between May 2019 and July 2019. The samples were obtained through masturbation into a sterile plastic container on-site and allowed to liquefy at 37°*C* after a 2–5 day period of ejaculatory abstinence, in accordance with the WHO guidelines (8). The inclusion criteria of the study were recruitment of patients between the age of 18 and 50 years who visited Rio de Janeiro semen bank for the purpose of infertility examination. Additionally, they must have given their consent to participate in the study and self-declared the absence of prior comorbidities or surgical history. Patients with azoospermia and severe oligospermia were excluded. The sample size calculation was based on the central limit theorem. The study was approved by the Ethics and Research Committee of the Municipal Health Secretary of Rio de Janeiro (SMS/RJ) under protocol number 3.291.691 and all participants provided written informed consent to participate in the study.

### Staining techniques:

To assess the sperm morphology, liquefied semen was mixed and dried smears were stained using either the Diff-Quick (Panótico Rápido® kit; Laborclin, Brazil) or Spermac (Spermac stain®; FertiPro N.V., Belgium) method. The staining procedures were performed according to the manufacturer’s instructions.

For the Diff-Quick method, slides were air-dried at room temperature, then fixed by immersing in a 0.1% triarylmethane solution for 5 *s*, followed by immersion in 0.1% xanthenes solution for 5 *s*, 0.1% thiazines solution for 5 *s*, and distilled water for 5 *s*. The slides were then air-dried at room temperature.

For the Spermac method, slides were air-dried at room temperature, then fixed by immersing in a formaldehyde solution for 5 *min*. The slides were air-dried, then stained by immersing for 1 *min* in solutions A, B, and C. Solution A was composed of ultrapure water, ethyl alcohol, rose Bengal, and neutral red. Solution B was composed of ultrapure water, ethyl alcohol, pyronin Y, orange G, and Phosphomolybdic acid. Solution C was composed of ultrapure water, janus green, and fast green FCF. The slides were washed in distilled water between each staining process (7 times). Finally, the slides were washed again and air-dried at room temperature.

### Sperm morphology analysis:

In this study, the morphological parameters of sperm were evaluated using blinded analyses by a senior embryologist using an optical microscope (Infinity Plus BM21100) under X1000 magnification implementing oil immersion technique. At least 100 sperm were evaluated for each sample. The spermatozoa were classified as having normal or abnormal morphology based on Tygerberg criteria (9), which include head defects (such as tapered, thin, large or small, multiple heads, abnormal acrosomal or post-acrosomal region), midpiece defects (such as cytoplasmic residues, thin and thick midpiece), and tail defects (such as bent, short, irregular, coiled, multiple, or no tail). The results were given in percentages for each classification, and the patient’s final diagnosis was determined by the lower reference limit of 4% normal sperm morphology, as established by the WHO manual (8).

### Statistical analysis:

The normality of distributions and homogeneity of variance of the staining techniques were evaluated using the Kolmogorov-Smirnov test. When the data showed a normal distribution, a paired t-test was conducted. In cases where the variables were not normally distributed, the Wilcoxon rank-sum test was performed. The final diagnosis was analyzed using the GLIMMIX procedure with a binomial distribution and Fisher’s exact test, using SAS SYSTEM 2000 (SAS Institute, Inc., USA). Statistical significance was defined as p≤0.05.

## Results

**Figure 1.**
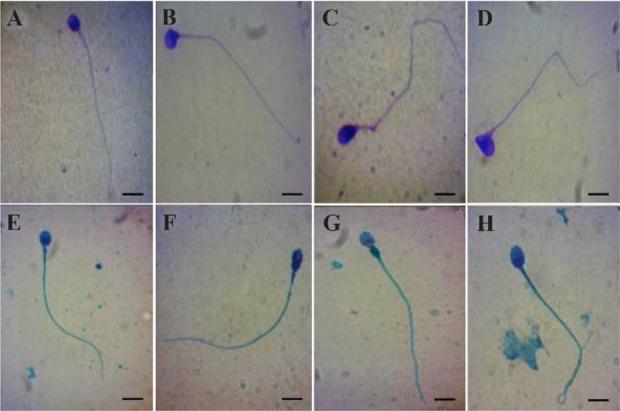
Spermatozoa stained with Diff-Quick (A–D) or Spermac (E–H). Representative photomicrographs showing normal spermatozoa (A and E), defect in the head (B and F), defect in the midpiece (C and G), and defect in the tail (D and H). Bar = 5 *mm*

Using Spermac stain resulted in better contrast between the midpiece and tail region compared to Diff-Quick stain. However, there was no significant difference in head morphology between the two stains. The analysis of sperm morphology using Diff-Quick resulted in a higher number of normal sperm and a lower number of midpiece abnormalities compared to Spermac. However, there was no significant difference in the head and tail abnormalities between the two stains. Despite the difference in the number of morphologically normal sperm, the final result was found to be statistically similar between Diff-Quick and Spermac using Tukey test and Fisher’s exact test (Figure 1 and Table 1).

**Table 1.** Summary of statistics of morphology evaluation in samples stained with Diff-Quick or Spermac

**Parameter**	**Diff-Quick stain**			**Spermac stain**			**Differences between Diff-Quick and Spermac staining (p-values)**

	**Mean**	**SEM**	**Median**	**Mean**	**SEM**	**Median**	
**Normal morphology**	3.98	0.41	4.0	2.8	0.33	2.0	0.0385
**Head defects**	93.42	0.66	94.0	94.24	0.61	95.0	0.3665
**Midpiece defects**	24.82	2.05	25.5	55.74	2.06	54.5	<0.0001
**Tail defects**	16.6	1.34	15.5	14.84	1.39	13.0	0.3032

Abbreviations: Standard error of the mean (SEM)

## Discussion

The Diff-Quick staining method was initially utilized for hematological examinations and has been found to be comparable to the standard method known as Papanicolaou (10–12). The Spermac staining method, on the other hand, was first used for seminal evaluations in domestic animals and has since been applied to a range of species including goats, horses, bulls, dogs, boars, and humans (13, 14). A study on cat spermatozoa demonstrated that this method provides a clear view of cell morphology, particularly the acrosome (14). In addition, this technique uses different colors to stain various parts of spermatozoa, facilitating a more comprehensive evaluation of human sperm morphology (15).

In the current study, when comparing the staining methods of Diff-Quick and Spermac, it was revealed that while both staining techniques showed similar results for sperm head morphology and tail morphology criteria, there were differences in the classification of normal and midpiece abnormalities. The percentage of spermatozoa with midpiece abnormalities was found to be higher with the use of Spermac stain. This difference may be attributed to the enhanced visualization of the midpiece provided by this stain. The limits of the midpiece were more clearly defined, thus allowing for greater accuracy in identifying morphological abnormalities in this region. Conversely, Diff-Quick stain did not provide clear delimitation of the midpiece, particularly its thickness. The advantage of Spermac in defining the midpiece transition between the distal portion and the proximal portion of the sperm tail was not observed in Diff-Quick stain, where the midpiece and tail appeared to be a single continuous structure.

In the assessment of sperm quality, it is essential to have a clear understanding of the morphological features of spermatozoa. This requires that various regions of the sperm be well defined, allowing for accurate measurement of length and thickness. As a result, it is possible that some midpiece abnormalities may have been underestimated in the Diff-Quick staining technique. This is related to the fact that Spermac staining method provides better visualization of the midpiece, clearly defining its limits, which makes it easier to identify morphological abnormalities in this region. Conversely, the Diff-Quick stain method does not provide clear delimitation of the mid-piece, leading to the appearance of a single structure that is continuous with the tail. As a result, the number of spermatozoa diagnosed as normal using Diff-Quick stain was higher compared to Spermac stain, while a higher number of spermatozoa with midpiece abnormalities was observed with the latter. This is significant because mid-piece alterations are closely associated with poor sperm motility and are considered a predictor of fertility (16).

The criteria for the final diagnosis are in accordance with the guidelines provided by the WHO manual (8). According to the manual, the diagnosis of teratospermia is established when less than 4% of the sperm are considered normal-shaped, while the diagnosis of normal morphology is established when 4% or more of the sperm are considered normal-shaped. When comparing Diff-Quick and Spermac, despite a significant difference in the number of sperm with normal shape, both results confirm the diagnosis of teratospermia. However, the average value for Diff-Quick is close to the cut-off point between different diagnoses, highlighting the need for further discussion on the appropriate cut-off point to be used across different staining methods.

In the realm of male infertility, it is widely recognized that the morphological examination of sperm plays a crucial role in identifying potential problems and contributing to the diagnosis of various fertility issues. The midpiece region of the sperm in particular is of utmost importance and a thorough evaluation of this region is often necessary in order to make an accurate diagnosis. Teratozoospermia, a condition characterized by abnormal sperm shape, can be caused by various factors, including dysplasia of the fibrous sheath, resulting in deformities in the midpiece and tail, leading to infertility. It is imperative that the examination of sperm morphology be conducted with utmost attention to details, particularly in the evaluation of the midpiece, in order to provide a comprehensive diagnosis and inform appropriate treatment options (15).

In addition to the deformities in the midpiece being associated with infertility, it has been shown that these anomalies can also result from an abnormal functioning of the centrosome. The centrosome is a critical component in cell division and any disruption in its function can severely impact the fertilization process and, in turn, the likelihood of infertility (17, 18). Furthermore, the selection of sperm with morphologically straight midpieces can enhance the centrosomal function of sperm and, as a result, elevate the success rates of fertilization through intracytoplasmic sperm injection (18). This highlights the importance of evaluating the midpiece of sperm during the fertility assessment process and its potential impact on the outcomes of assisted reproductive techniques. The two staining methods used in this study have their own advantages and disadvantages. One of the key advantages of Diff-Quick stain is its lower cost and its quicker application, taking only approximately 20 *s* compared to approximately 8 *min* required for Spermac method, excluding air-drying steps. This makes Diff-Quick a more practical option for laboratory use, but it overlooks details in the evaluation of spermatozoa structures, which is where Spermac excels, particularly when a more in-depth analysis of the sperm morphology is needed. Furthermore, to the best of our knowledge, this is the first report demonstrating a significant difference between the alterations in the midpiece.

## Conclusion

The findings of the current study show that Diff-Quick staining results in a higher mean value for normal spermatozoa classification, which can be attributed to the evaluation of the sperm midpiece. This difference between the two staining methods has a crucial impact on the final diagnosis of patients, particularly in the field of male fertility. While sperm morphology is an important factor in the evaluation of male fertility, these findings raise the question of whether a single cut-off point for all staining methods is appropriate. Despite not being recommended by the World Health Organization manual (1, 8), the Spermac method has the potential capacity in detecting midpiece alterations in patients, which could enhance the chances of success in in vitro fertilization.

It is important to note that these results have significant implications for clinical practice and future research in the field of male infertility. However, further studies are required to explore the effectiveness of different staining methods and to determine the optimal cut-off points for each method to ensure accurate diagnoses and treatments for patients.
